# Fragmentation Follows Structure: Top-Down Mass Spectrometry Elucidates the Topology of Engineered Cystine-Knot Miniproteins

**DOI:** 10.1371/journal.pone.0108626

**Published:** 2014-10-10

**Authors:** Michael Reinwarth, Olga Avrutina, Sebastian Fabritz, Harald Kolmar

**Affiliations:** 1 Institute of Organic Chemistry and Biochemistry, Technische Universität Darmstadt, Darmstadt, Germany; 2 AB Sciex Germany GmbH, Darmstadt, Germany; Johns Hopkins University, United States of America

## Abstract

Over the last decades the field of pharmaceutically relevant peptides has enormously expanded. Among them, several peptide families exist that contain three or more disulfide bonds. In this context, elucidation of the disulfide patterns is extremely important as these motifs are often prerequisites for folding, stability, and activity. An example of this structure-determining pattern is a cystine knot which comprises three constrained disulfide bonds and represents a core element in a vast number of mechanically interlocked peptidic structures possessing different biological activities. Herein, we present our studies on disulfide pattern determination and structure elucidation of cystine-knot miniproteins derived from *Momordica cochinchinensis* peptide MCoTI-II, which act as potent inhibitors of human matriptase-1. A top-down mass spectrometric analysis of the oxidised and bioactive peptides is described. Following the detailed sequencing of the peptide backbone, interpretation of the MS^3^ spectra allowed for the verification of the knotted topology of the examined miniproteins. Moreover, we found that the fragmentation pattern depends on the knottin’s folding state, hence, tertiary structure, which to our knowledge has not been described for a top-down MS approach before.

## Introduction

Precise information concerning identity, structure, and pharmacokinetics of drug candidates is an important issue in the development of biopharmaceuticals, among them a vast number of bioactive peptides [1]. Therefore, reliable and reproducible analysis of their structure and topology needs well-elaborated high-throughput methods. Tandem mass spectrometry (MS/MS or MS^2^) has become a valuable tool for the identification and quantification of peptides and proteins. Recent experiments indicate that MS analysis can also be applied to their structural characterization since folded and unfolded molecules may give rise to different fragmentation patterns upon ionization [1]–[3]. Moreover, MS^2^-based methods can be applied to identify and characterize inter- and intramolecular disulfide bonds along with NMR and X-ray analysis [2]. Following the bottom-up methodology for the determination of disulfide-bond topologies in multi-disulfide proteins, the analytes are partially reduced and subjected to enzymatic digestion prior to the analysis *via* MS or MS/MS [4]–[6]. Although this approach provides decisive information on the primary structure, determination of disulfide connectivities *via* MS analysis of proteolytic fragments of non-reduced or partially reduced peptides with high disulfide content remains a complicated task.

In some instances, the available specific enzymatic cleavage sites within the peptide of interest do not necessarily lead to one-cystine-one-peptide fragment distributions after digestion of folded species, or no appropriate cleavage sites are present at all. Moreover, partial reduction and S-alkylation prior to proteolysis often results in a complex mixture of variants [4], [7], [8]. Furthermore, lack of information originating from incomplete sequence coverage and the fact that not every posttranslational modification (PTM) could be detected, are additional disadvantages [4], [5], [9]–[15].

**Figure 1:**
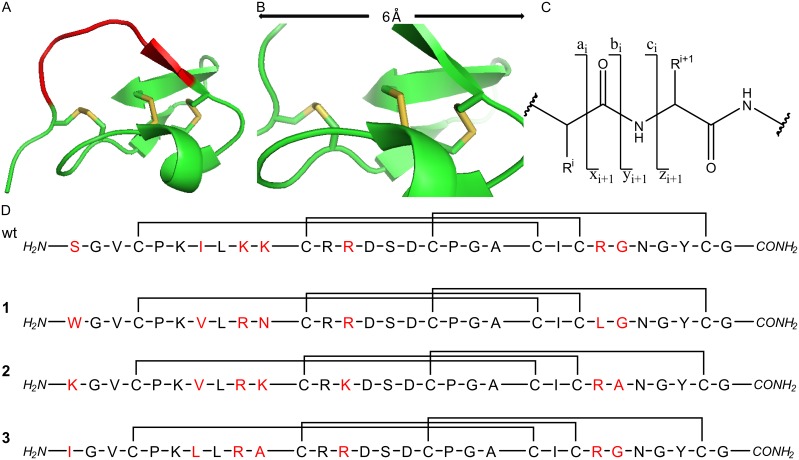
Structure of synthetic open-chain MCoTI, structural overview and fragment ion formation. (**A**) 3D structure of synthetic open-chain cystine knot oMCoTI (pdb: 2IT8).15 Active loop is shown in red. (**B**) A 6 Å close-up on the disulfide-tightened core of MCoTI. For both (**A**) and (**B**) disulfides are shown in yellow. (**C**) Overview of the generation of peptidic fragment ions upon CID. (**D**) Sequences of the parent MCoTI wild type (wt) and the miniproteins (**1–3**) used in this study. Positions with altered amino acid residues (regarding the wild type sequence) are marked red.

In recent years, efforts have been made to apply a top-down methodology for the characterization of full-length native proteins and peptides *via* concurrent cleavage of disulfide bonds using MS/MS methods minimizing loss of information [8], [11]–[14], [16]–[18]. To this end, different MS fragmentation methods were applied, among them the widely employed collision-induced dissociation (CID) featuring low collision energies. Electron-transfer or capture dissociation (ETD or ECD, respectively) methods have also been used for the generation of MS/MS spectra [8], [17]–[21]. For CID the mobile proton theory, which states that positive charges are randomly distributed among all amino acids of an analyzed peptide, thus facilitating amide N-protonation and cleavage, is an important tool to interprete MS spectra [16], [22]–[27]. Regarding arginine-rich peptides this is not entirely true since the basic side chains are known to sequester charges [16], [22]–[24]. Thus, the ratio of charge to the number of arginines within the sequence must be over one to make the mobile proton theory applicable [16], [22]–[24]. Hence, the overall charge of the peptide plays an important role since protonated arginine side chains are known to promote disulfide cleavage [15], [19]–[24]. As a consequence, the collision energy required for the cleavage of disulfides depends on the ratio of cystine to arginine units within the sequence [16], [22]–[24]. Additional difficulties upon assigning peptidic fragments in the complex spectra progressively increase with the size of the peptide and the number of S-S bonds [8], [17], [18]. The side chains of several amino acids tend to neutral losses and the formation of uncommon product ions under ionization conditions due to specific proximity effects [2], [8], [17], [18], [28]. In particular, asymmetric cleavage of disulfide bonds results in the formation of both perthiocysteine (Ptc) and dehydroalanine (Dha), of which the latter is known to induce cleavage of the peptide backbone not at the amide bond, but promotes fragmentation between the amide nitrogen and the α-carbon, hence forming c-ions (Figure 1C) [2], [8], [16], [28], [29]. These effects lead to an increased number of signals in the spectrum resulting in a statistically high chance of false-positive assignments. Thus, regarding large disulfide-rich peptides and proteins, the gained spectra require further in-depth analysis due to their complexity [30].

Following previous studies on the analysis of cysteine-rich proteins, Green and co-workers elaborated the idea of an additional fragmentation stage (MS^3^) and used it for a top-down approach to analyze chicken lysozyme [2], [22], [31], [32]. Therein, nozzle-skimmer ionization was used as the first dissociation source, while regular CID served as a second fragmentation stage [2]. Although this pseudo MS^3^ approach has delivered promising results, the MS^3^ utilizing two dissociation sources could facilitate the detailed evaluation of the disulfide pattern topology.

As model disulfide-rich macromolecules for investigation of structural features and disulfide bond connectivities by application of MS^3^ methodologies, engineered cystine-knot protease inhibitors were used in the present study. These bioactive peptides, also known as knottins, consist of about 30 amino acid residues and are characterized by a unique, ‘pseudo-knotted’ architecture [33]–[35].

Knottin’s structural core is defined by three β-strands which are interconnected by three disulfide bonds. The bonds between CysII and CysV as well as CysI and CysIV form a ring which is penetrated by a third cystine, connecting CysIII and CysVI (Figure 1B) [34]–[36]. Additional thermodynamic stability is provided *via* an extensive network of hydrogen bonds between the peptide backbone and the side chains of the amino acids located within the β–sheets [37], [38]. Therefore peptides containing this structural motif display an exceptional structural, thermal, and biological robustness [35], [39], [40].

Knottins are considered excellent scaffolds for the generation of tailor-made compounds for diagnostic and therapeutic applications since the surface-exposed loops consist of segments with high structural and numeral flexibility, while the conserved core only tolerates minor amino acid exchanges [32], [35], [41]–[43]. Engineered miniproteins derived from trypsin inhibitors of the bitter gourd *Momordica cochinchinensis* (MCoTI) have been successfully applied for the inhibition of proteinases of clinical relevance, among them human mast cell tryptase β, foot-and-mouth-disease virus 3C protease, and, most recently, cancer-related protease matriptase-1 (Figure 1D) [44]–[46]. Moreover, a cystine-knot peptide from cone snails, Ziconotide, has been approved for the treatment of severe and chronic pain [47]. Although optimized conditions for both chemical and recombinant synthesis of these miniproteins have been recently reported, oxidative folding towards the respective bioactive isomer still reveals no information on the disulfide topology [7], [48], [49]. Biological activity is an important indication for a correct fold, but for engineered variants with multiple amino acid exchanges this information may not necessarily prove the knotted cystine pattern [45].

To date, determination of disulfide bond connectivities for this class of peptides relies on NMR and X-Ray of native peptides as well as on MS analysis of chemically or enzymatically modified ones [17], [18]. Compared to the NMR and X-Ray technology, a major advantage of an analytical technique such as a mass spectrometry is the requirement of less analyte. Despite the fact that method development may be time consuming, the broad availability of MS instruments enable more laboratories to make structural determinations and, when using well-established methods, analysis can be performed in shorter time. Thus, Tam and coworkers recently described disulfide mapping of the cyclotide hedyotide B2 by MS^2^ analysis of the partially reduced and alkylated peptide [50]. Furthermore, Balaram et al. elucidated disulfide topology of four-cysteine native peptides as well as enzymatically treated conotoxines [17]. Camadro *et al*. described structural analysis of knotted Psalmopeotoxin I through combined tandem MS and ^15^N^–^NMR [18]. In the present study we applied MS^3^ technology for the characterization of folded and oxidised open-chain MCoTI variants. For the confirmation of structural and topological characteristics we employed a top-down approach to deliver information on elements regarding the primary, secondary, and tertiary structure of the miniprotein.

## Materials and Methods

Peptides **1**, **2**, and **3** have been synthesized as previously reported [44], [49].

Detailed information on mass spectrometric measurements is provided in the supporting information (Method S1). Briefly, the 4000 QTRAP LC/MS/MS and the 6500 QTRAP LC/MS/MS systems (AB Sciex Germany GmbH, Table S1) were used. For MS full scans experiments signal intensity was adjusted to achieve 1e6 counts per second (cps) maximal signal intensity at 10 µL per minute infusion from a syringe pump. Peptide stock solutions have been prepared by dissolving 0.5 mg of the respective peptide in 1 mL of a mixture of 20% methanol (Fluka Analytical, LC-MS CHROMASOLV) and 80% water (Merck KGaA, LC-MS LiChrosolv) with 0.2% formic acid (Fluka Analytical, analytic additive).

For MS^2^ experiments both declustering potential (DP) and collision energy (CE) have been optimized *via* ramping. Regarding MS^3^ measurements, resonance excitation energy (AF2) was additionally optimized (using ‘ramping’ functionality of Analyst software). All spectra were accumulated to ensure distinct differentiation between signals and noise.

## Results

### Experimental design and general results

As model peptides we chose variants derived from the cystine-knot peptide MCoTI (Figure 1), a synthetic protease inhibitor combining the three-disulfide pattern with the open-chain amide backbone [44]. Since peptides of this family are known for their structural stability regarding biological and chemical decomposition, denaturation or disulfide scrambling should not take place under acidic conditions which were used for mass-spectrometric analysis [7]. The examined miniprotein variants comprised selected exchanges within the flexible loops [44], [49].

**Figure 2:**
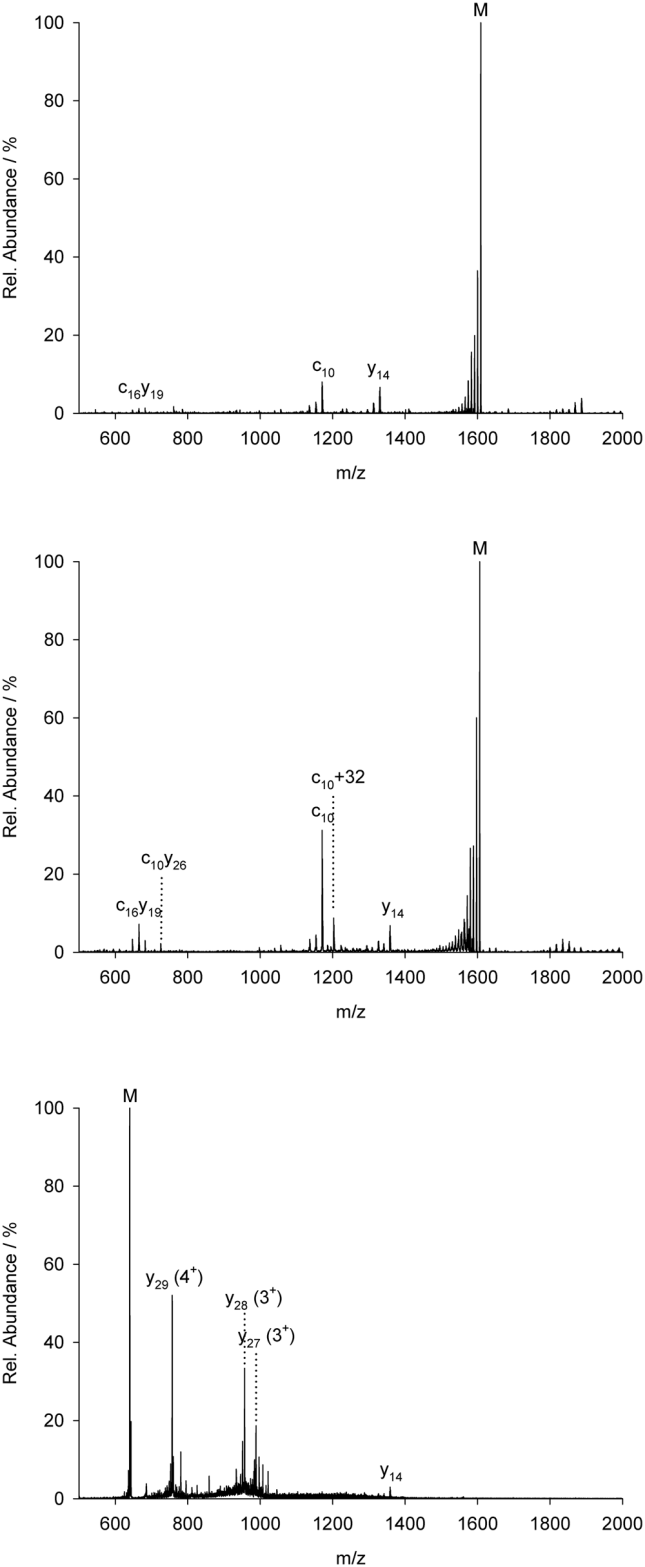
MS^2^ of MCoTI peptide 1. (**A**) CID of the doubly charged ion of reduced peptide 1. (**B**) CID of the doubly charged ion of folded miniprotein. (**C**) CID of the five-fold charged ion of folded miniprotein.

To investigate the influence of structural characteristics on the fragmentation pattern, tandem mass spectrometric analysis triple quadrupole systems with a linear ion trap were used (ESI, Table S1). The design of these systems allows for two dissociation steps upon selection of the respective precursor ions. Thus, we applied CID to both the oxidised three-disulfide peptide 1 (Figure 1) and its reduced precursor. In the resulting spectra, the unique peaks and those showing the typical shift of hydrogens or sulfhydryl groups (Figure 2 and Figures S1–S8) were compared.

Identification of the respective fragment was achieved *via* selection of the corresponding precursor for MS^3^ (Figure 2 and Figures S9–S13). Ion path parameters and ionization energies, namely the declustering potential, the collision energy and the excitation energy for the MS^3^ were semi-automatically optimized (ramped) towards maximum intensity of the respective product ions. Similar fragmentation patterns were found for peptides 1, 2, and 3 that deviated from each other in the amino acid sequences of loop 1, 2, and 5 (Figure 1) [44].

**Figure 3:**
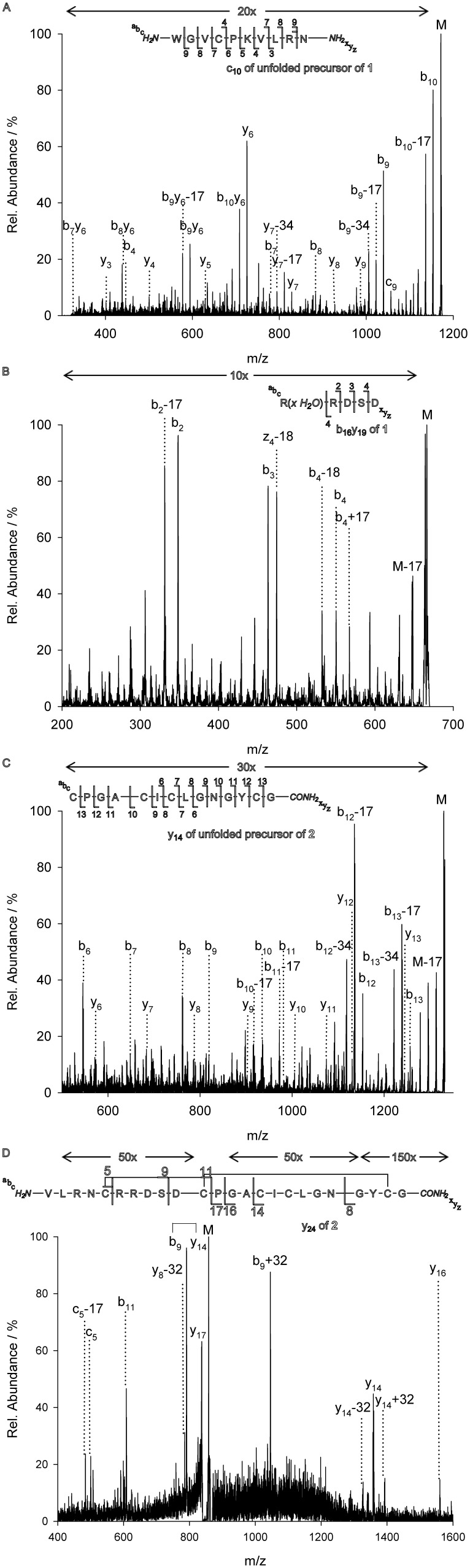
MS^3^ of the CID-obtained major fragments of MCoTI 1. (**A**) MS^3^ of c_10_ of the doubly charged ions of unfolded peptide. (**B**) MS^3^ of c_16_y_19_ of the doubly-charged ions of folded miniprotein. (**C**) MS^3^ of y_14_ of the doubly charged ions of unfolded peptide. (**D**) MS^3^ of y_24_ of the five-fold charged ions of folded miniprotein. Arrows above the spectra indicate intensity amplifications.

While amino acid exchanges outside the inhibitory loop 1 that protrudes into the active site of the respective protease induced no altered fragmentation of the native peptide, as expected, relative intensities of the respective ions dropped upon arginine exchanges since protonated arginine side chains are known to promote disulfide cleavage [15], [19]–[21]. Indeed, the relative peak intensity of the C-terminal fragments (y_14_, Figure 2 and Figures S7 and S8) is higher for the arginine-containing variants (**2** and **3**) compared to **1** that lacks this moiety. In contrast, for the fragment c_16_y_19_ or c_16_z_20_, respectively, only a minor decrease of signal intensity was observed for peptide **3**, since it still contained an additional arginine which was kept unchanged in each variant. Moreover, signal intensity of product ion series immediately drops upon cleavage of arginine, which is caused by the sequestered charge at the arginine side chains, thus decreasing the intensity of the following b-ions by leaving the majority of them neutral (Figures 3B and Figure S9).

The MS^2^ spectra only showed minor differences between the unfolded and folded variants (Figure 2). Indeed, the fragmentation patterns were similar, apart from the expected unsymmetrical cystine dissociations resulting in Ptc or Dha moieties and different intensities for the fragments c_16_y_19_ (c_16_z_20_) and, particularly, c_10_y_26_.

CID spectra obtained from the lower charged precursors [M+2H]^2+^ or [M+3H]^3+^, respectively, were significantly enriched with three fragment species (c_10_, c_16_y_19_ and y_14_) covering the whole sequence (Figure 2). These major fragments were identified and sequenced using MS^3^ methodology. This pattern lacked Cys11, which was caused by the formation of the major c-ion fragment c_10_ (Figure 2). C-ion formation has been reported to occur upon cysteine fragmentation towards Dha [2].

For high-charged states, e.g. [M+5H]^5+^, MS^2^ spectra of the folded miniprotein displayed a different fragmentation pattern. Interestingly, we observed formation of several b-ions which could not be detected upon low-charge CID (Figure 2). While all b-ions comprising large linear fragments still possessed at least one disulfide, one fragment was identified lacking the central SD motif, thus resulting in the generation of a ‘bridged’ fragment (Figure 3D). However, assignment of the fragments was complicated by the multiple charge states within the spectra.

### Elucidation of primary structure

For the sequential analysis, the major fragment ions c_10_, c_16_y_19_ and y_14_ were chosen for MS^3^ analysis. Assignment of b- and y-ions was complicated since several obviously predetermined breaking points dictated the shape of the spectra, while other fragments were hard to distinguish from noise (Figure 3). Additionally, ‘satellite’ peaks were observed for each sub-fragment ion, originated from neutral losses of amino acid side chains, e. g. lysine (−17 Da) [51].

In the case of the aminoterminal fragment c_10_, intensity of b- and c-ions decreased upon cleavage of Arg9 (which is equivalent to the formation of the b_9_/c_9_ ion), due to the sequestered charge at the guanidyl moiety (Figure 3A). Regarding the y-ions, the dominating fragment in the MS^3^ spectrum was the y_6_-ion which became the starting point for further b- ion fragmentation leading to the generation of b_10_y_6_, b_9_y_6_, b_8_y_6_, and b_7_y_6_ ions (Figure 3A). Moreover, the y-ion series was detected until formation of y_3_.

The c_16_y_19_, respectively, c_16_z_20_, fragment was analyzed by MS^3^ and sequencing revealed its water adduct. For this fragment we observed b_4_ as well as y_4_, y_3_, and y_2_-subfragments which provided detailed sequence information (Figure 3B). As expected, the spectrum was enriched by y-ions formed upon aspartate cleavages. Interestingly, the b_4_ ion fragment was not observed as water adduct anymore, thus indicating Arg12 being primarily responsible for the binding of water (Figure 3B).

The carboxyterminal fragment y_14_ delivered complete information regarding the primary structure. B-ion formation from b_13_ to b_6_, as well as y-ion formation of y_13_ to y_6_ (Figure 3C) was observed. However, this spectrum was also complicated by predetermined breaking points as well as neutral losses, due to several proximity effects or directed non-standard fragmentation like Dha-promoted c-ion formation.

### Secondary structure

Determination of the cystine connections could not be achieved directly through detection of ‘bridged’ fragments for lower-charge states. This is due to the fact that the cysteine residues are close to each other within the sequence, e. g. having only one isoleucine in between CysIV and CysV. Moreover, analysis is complicated by the intrinsic knotted structure and the fact that disulfide dissociation occurs upon charge localization on basic side chains [8], [17], [18].

**Figure 4:**
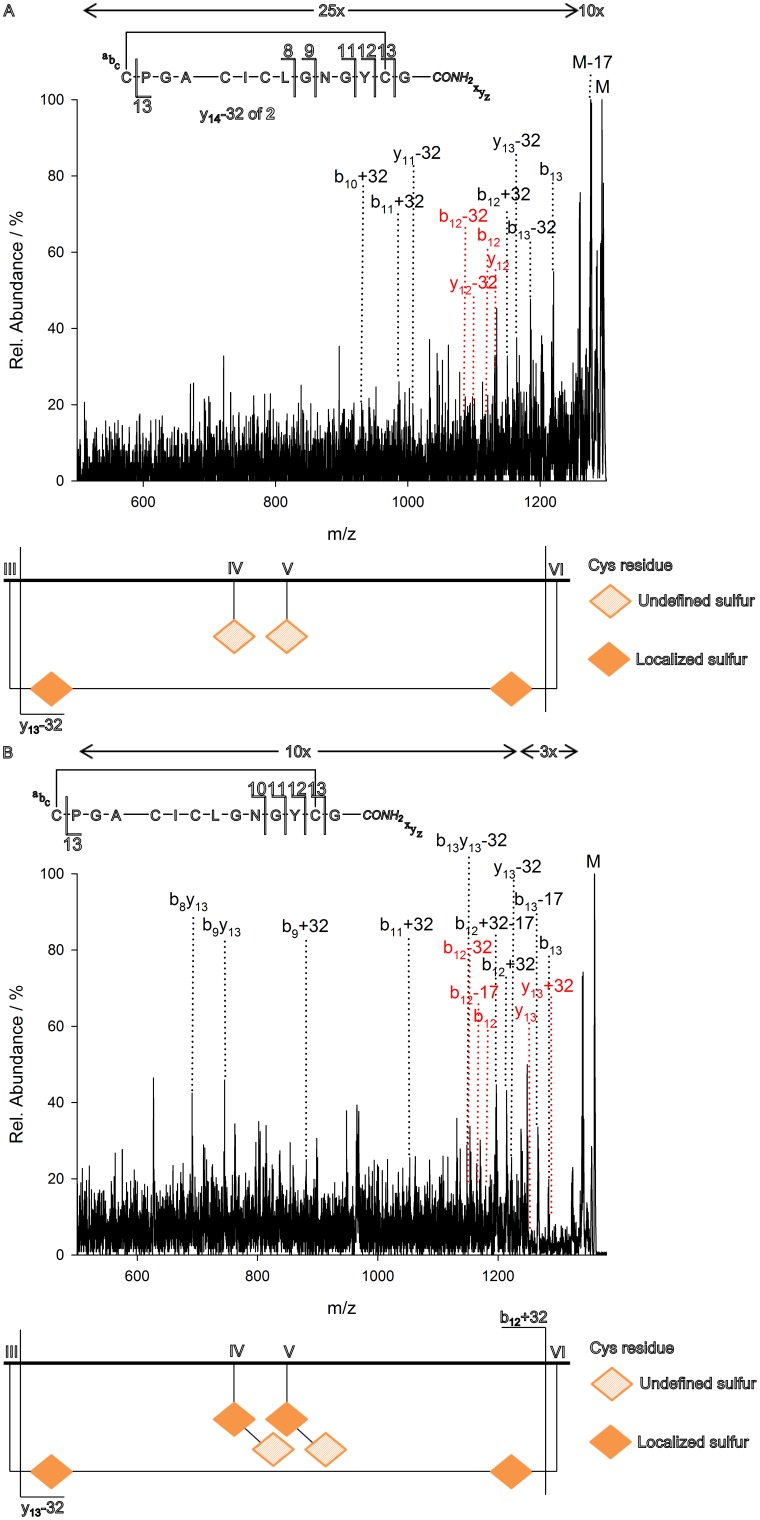
MS^3^ of y_14_-32 (A) and y_14_+32 (B) and the resulting combinatorial interpretation. In red are inexistent peaks to provide evidence on the respective Ptc or Dha cleavage. Arrows above the spectra indicate intensity amplifications.

For this reason the four-cysteine fragment y_14_ was of particular interest as it contained an intact disulfide bridge. For the determination of the expected III–VI connection, we analyzed three related m/z fragments, namely, 1294, 1326 and 1358, *via* MS^3^. These ions correspond to a four-cysteine-three-sulfur (y_14_-32, Figure 4A), a four-cysteine-four-sulfur (y_14,_ Figure 3C) and a four-cysteine-five-sulfur (y_14_+32, Figure 4B) fragment, respectively. MS^3^ analysis of those fragments of **1** suffered from the absence of an arginine compared to **2**, thus these fragment series were displayed a higher intensity in the arginine-containing variant (Figures S11 and S12). Nevertheless, combinatorial interpretation of the resulting spectra clearly revealed the predicted connection of CysIII to CysVI (Figure 4). Involvement of CysIII was verified since it was found to be cut off exclusively as a Ptc moiety for all three parent fragments (Figure 4A), hence the remaining y ions were exclusively found as y-32 ions. After cleavage of the Ptc from the four-cysteine-three-sulfur fragment, a three-cysteine-one-sulfur one (y_13_-32, Figure 4A) was received, while the cleavage of Cys (y_13_) or Dha (y_13_+32) moieties did not occur (Figure 4A and Figures S11 and S12, red ‘peaks’). Indeed, the distribution of the sulfur atoms was exclusively found to be on CysIV and CysV. Hence, connections of IV–V, IV–VI, and V–VI could be excluded. As a result, the only possible intramolecular connection was the expected III–VI one. To provide further evidence of the connection CysIII to CysVI, analysis of the four-cysteine-five-sulfur fragment was performed. After generation of the b_12_ ion, a three-cysteine-three-sulfur fragment (b_12_+32, Figure 4B) was obtained, which is underpinned through the absence of the ions b_12_-32 and b_12_ (Figure 4B and Figures S11 and S12, red ‘peaks’). Thus, connections of III–IV and III–V could be excluded solely leaving III–VI one possible. Nonetheless, regarding the lower charged precursors, no exact conclusion could be made on the two remaining cystines, which still could be the native, knotted I–IV/II–V or the intuitively implausible, unknotted I–V/II–IV connection.

For verification of the knottin fold, the second disulfide connection was evaluated. However, the probability that a bridged fragment is formed upon CID is very low since at least three peptide bonds need to be cleaved while the respective cystine connection has to remain intact. For this purpose we focused on the higher charged states (5+) for the relationship between the protonated arginine residues and the fragmentation of disulfide bonds (see introduction section) [16], [23], [24].

**Figure 5:**
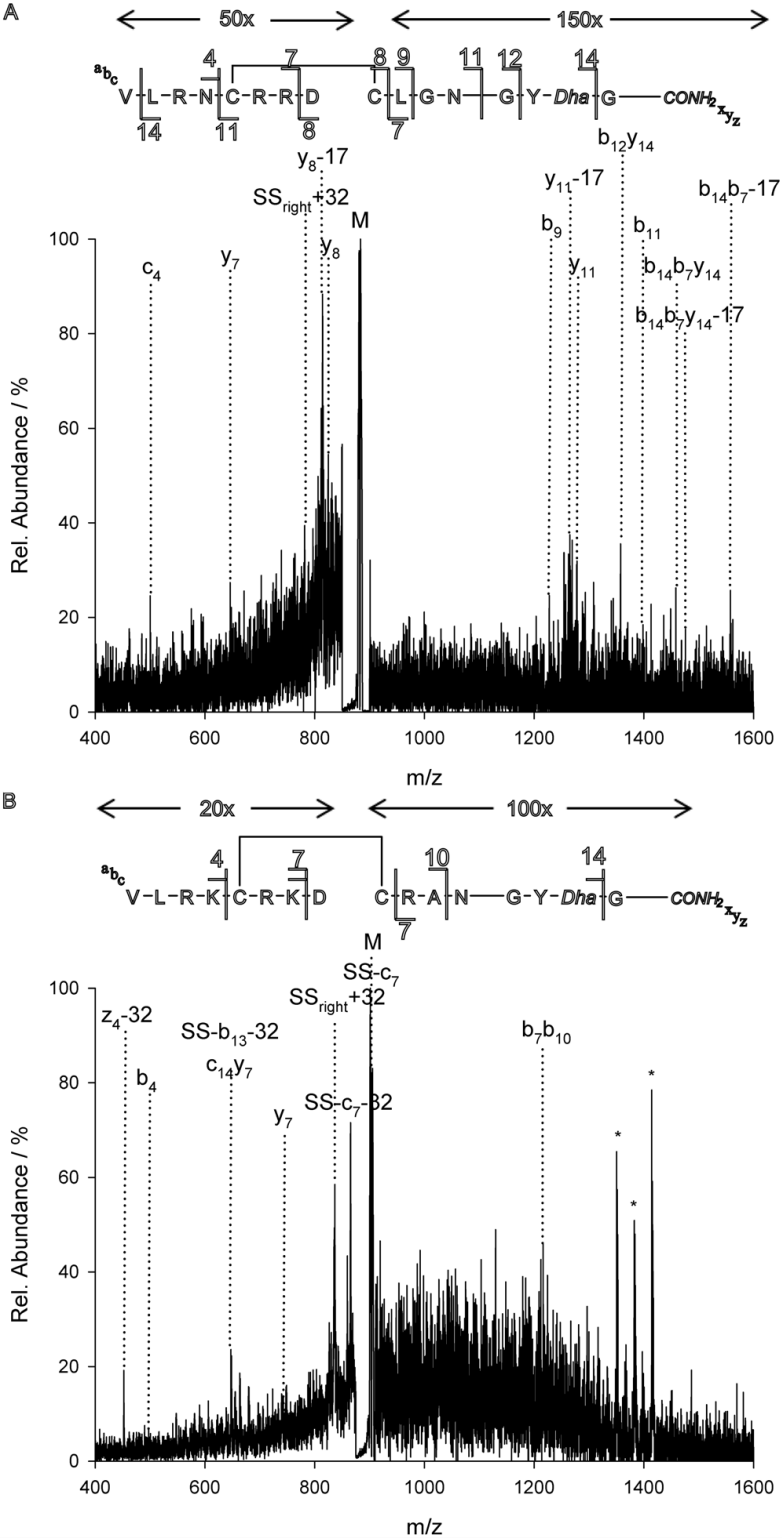
Spectra and assigned structures of the bridged fragments. (**A**) MS^3^ of the bridged fragment of **1**. (**B**) MS^3^ of the bridged fragment of **2**. Arrows above the spectra indicate intensity amplifications.

However, we found the linear y_24_ fragment containing the connections CysIII–CysVI as well as CysII–CysIV or CysII–CysV, respectively (Figure 3D). Upon cleavage of the labile SD motif, this fragment comprised two linear fragments connected through one intermolecular cystine. For the proof of the II–V connection, this fragment was subjected to MS^3^ analysis. This procedure revealed an ion consisting of two linear peptide chains connected through the disulfide linkage between CysII and CysV. Direct MS^3^ analysis of this bridged fragment produced several ions belonging to the expected product connected *via* the linkage of CysII and CysV (Figure 5). Since peak intensities were relatively low due to the unlikeliness of the multiple, specific breakages necessary for the generation of this fragment, the complete fragment series could not be detected although further optimizations of the detector parameters could increase the signal to noise ratio. Nevertheless, the major product ions were identified.

For peptide **2** this procedure of fragment analysis was interfered by another fragment ion with nearly identical mass (Figure 5), which might be avoided by tightening the isolation window of the precursor ions. Nevertheless, the expected product ion was verified, too. Additionally, fragmentation revealed a small fragment ion which was identified to originate from the II–V-connected peptide ion (Figure S13). This in-depth analysis was not performed for verification of peptide **3**.

### Tertiary structure

Comparison of the CID spectra of the positively charged folded and unfolded peptides revealed a distinct intensity increase of the peak corresponding to both the c_16_y_19_ as well as the c_10_y_26_ fragment for the folded miniprotein (Figure 2), which was confirmed by MS^3^ analysis. Generation of both fragments was compared for each miniprotein variant at different collision energies. Ratios depicted in Table 1 result from collision energies optimized towards a maximum TIC.

**Table 1:** Amount of loop ratios in CID of the respective peptides.

	Intensity of c_10_y_26_ (% of TIC)		Ratio	Intensity of c_16_y_19_ (% of TIC)		Ratio
	Reduced	Folded		Reduced	Folded	
**1** (CE: 70 V)	0.25	2.2	1∶9	1.1	7.2	1∶7
**2** (CE: 50 V)	0.17	3.2	1∶19	<0.1	1.8	1∶18
**3** (CE: 85 V)	3.5	21.5	1∶6	1.8	9.8	1∶5

**Figure 6:**
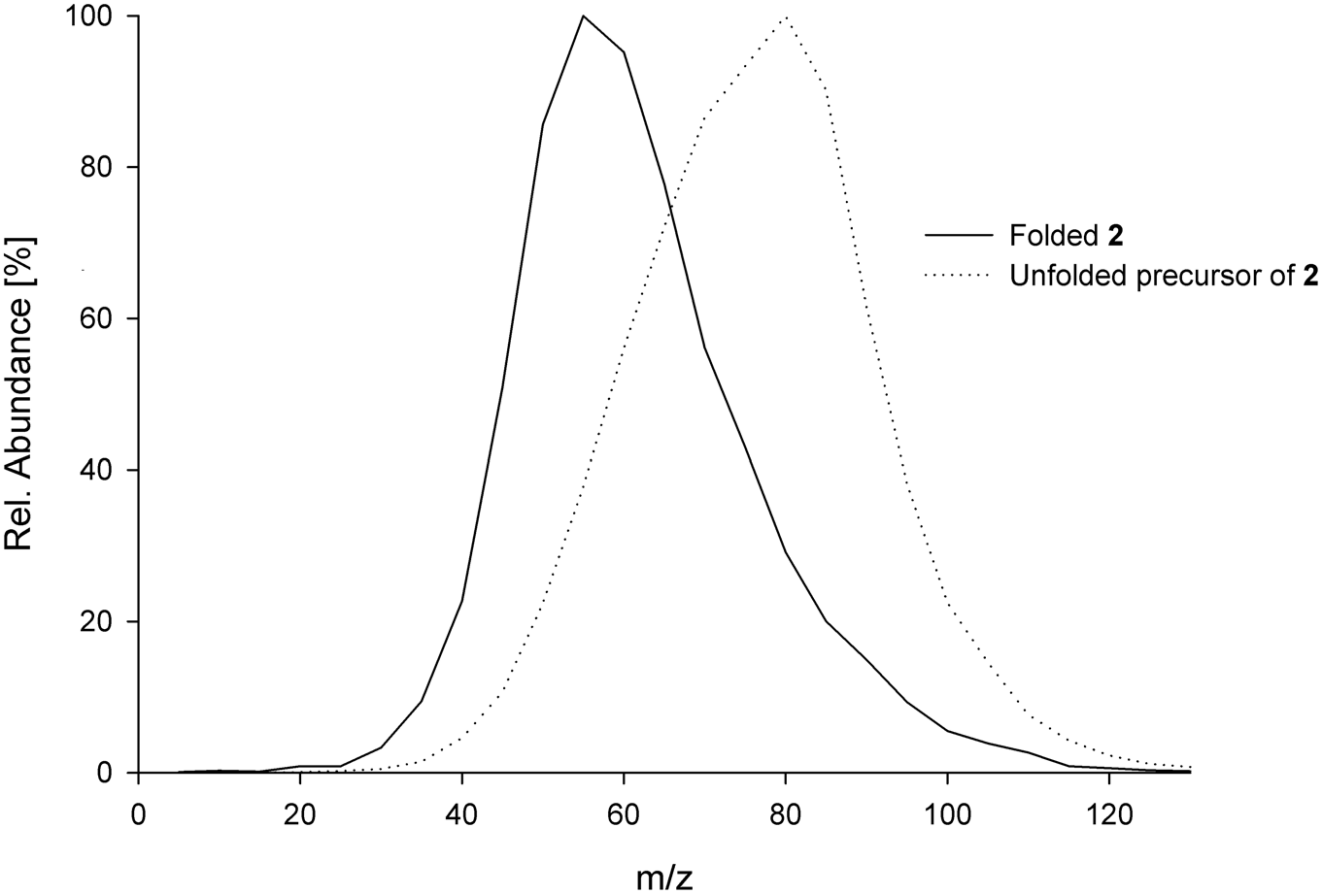
Optimization of the collision energy towards a maximum formation of fragment c_10_y_26_ of 2.

For miniproteins **1** and **3** ratios of over 6 regarding the formation of both fragments for the reduced peptide and the folded knottin were observed. For miniprotein **2** the observed ratio was approximately 20. Additionally, the dependency of these ratios as well as the maximum fragment formation on the applied collision energy in obedience to the folding state was elucidated. Interestingly, for **1** and **3** no influence of the folding state on the maximum fragment formation was observed. For miniprotein **2**, instead, increasing collision energies lead to an adjustment in fragment formation. Thus, a semi-automatic optimization of the collision energy to determine the energy with a maximum formation of fragment c_10_y_26_ (Figure 6) was performed. Maxima at different collision energies were observed for the reduced peptide and the folded knottin. This accounted for high ratios at CE 50 as well as decreasing ratios for increasing collision energies.

## Discussion

The analyzed miniprotein variants displayed very similar fragmentation patterns, notwithstanding the fact that sequence alterations have been applied for all sequential and structural regions (Figure 1C). Interestingly, the resulting fragmentation pattern was not influenced by sequential changes. Therefore, the reproducibility of the results in this experimental setting should allow for the prediction of CID spectra for novel representatives of the MCoTI family.

Fragmentation was obviously guided by cysteines as well as aspartates. In non-mobile proton systems aspartate side chains are known to induce carboxyterminal cleavage [2], [16]. Hence, predetermined cleavage sites within the “DSD” motif of the respective peptide variants were verified. Regarding the cysteine residues, the fragmentation pattern was predetermined by CysII and CysIII of the sequence, although the folding state, hence secondary and tertiary structural elements, only had influence on signal intensity rather than fragment formation itself. This indicates a cysteine-induced cleavage of the peptide backbone resulting in the formation of c-ions for both reduced and oxidised cysteines [2], [16]. In contrast, the charge state played a key role in fragmentation. Higher charged precursors led to the formation of large b ion fragments. As a consequence, only a decreased amount of cysteine-guided fragmentation could be observed. Both findings strongly correlate with the fragmentation patterns reported by Green and co-workers [2], [16].

We achieved full sequence coverage for the reduced and oxidised peptides in this top-down approach. For all three peptides similar fragments were detectable and could be identified. Hence, for evidence of known sequences or those with minor exchanges, sequence analysis can be performed easily without sophisticated methods of sample preparation or spectra analysis.

Disulfide topology is the major determinant of secondary structure for cystine-knot miniproteins. Since no predetermined breaking points resulting in ‘bridged’ fragments were found in CID-based spectra, in-depth analysis towards determination of the cystine pattern was performed for relatively large, linear fragments containing intramolecular disulfides. MS^3^-based sequencing was performed and combinatorial interpretation of the resulting spectra revealed the expected disulfide connectivity (Figure 4). This analysis was based on the generation of Dha or Ptc moieties upon CID resulting from unsymmetrical breakage of cystines. This untypical manner of analysis has rarely been reported for the topology determination in a top-down approach since it is hardly applicable for large proteins [17], [18], [52]. The specific restrictions present for cystine-knot miniproteins, in particular, the short sequential distances between the respective cysteine residues and the overlap of cystine connections, almost exclude the generation of ‘bridged’ fragments which are not connected *via* the amide backbone and thus the exact determination of disulfide patterns [17], [18].

Interpretation of the spectra of the bridged fragments for the determination of a second cystine connection was complicated through their complex nature and low intensities, thus intensive manual analysis was required. Since multiple collisions are possible within the collision cell and the linear ion trap, the analyzed fragments indeed most likely were *tertiary* or *quaternary* fragment ions. Moreover, probabilities for the generation of those multiple fragmentation events are relatively low, peak intensities of the resulting ‘pseudo-MS^n^’ spectra are very low as well. This only allowed for detection of sequentially predetermined breaking points, thus no formation of fragment ion series was observed. Instead, several individual species with increased intensities were determined and the bridged fragments could be clearly identified for both **1** and **2**, although the spectra of **2** were interfered by another fragment ion with a similar mass (Figure 5). Despite this complicated methodology for the *ab initio* determination of disulfide patterns, verification of knotted disulfide connectivity of oxidised peptides of the MCoTI family now can be routinely applied without time-consuming methods by fragment analysis of common CID spectra.

We observed increased intensities of the fragments corresponding to the surface-exposed loops in the CID spectra of the oxidatively folded miniproteins. Since some loops of knottins of the MCoTI family are subjected to tensed conformations upon oxidative folding, their increased energetic content may account for the increased peak intensities (Table 1). This is particularly reasonable for loop 1 (flanked by CysI and CysII) as its biological function, i.e. binding to the respective protease, strongly depends on the folding state of the peptide, which requires the correct disulfide pattern along with the surface-exposed and tensed inhibitory loop region [53]. Accordingly, these elements of the tertiary structure most likely account for the rise in peak intensity.

Further interest was spent on the investigation of the shifted maxima in collision energy regarding the formation of fragment c_10_y_26_ of reduced and folded **2**. Interestingly, peptides **1** and **3** did not show this behavior. The additional basic amino acid Lys10 in the inhibitory loop 1 as well as a slightly different 3D structure may account for this interesting characteristic of **2**. Applying this type of analysis, determination of the correct cystine-knot fold of miniproteins, at least with sequences similar to **1**, can now be achieved through comparison of fragment formation in the folded and unfolded state. MS-based detection of the influence of chemically modified amino acids on the tertiary structure of proteins has often been reported, but to the best of our knowledge, an effect of the tertiary structure on the fragmentation pattern has not been described for a top-down approach before [1], [8], [54]–[56].

### Conclusion

In this study a set of synthetic matriptase-1 inhibitors were analyzed through MS^3^ methodology. Full sequence coverage and determination of disulfide topology were achieved. Determination of primary structure is of particular interest for ‘one-bead-one-compound’ libraries which have already been applied for linear peptides and for medium throughput screening of bioactive molecules [57], [58]. It would be interesting to see whether this methodology can be applied to the on-bead sequence analysis of cystine-knot peptides although oxidative folding of the resin-bound peptides is still critical [59]. Additionally, an influence of secondary and tertiary structure on the fragmentation pattern was observed. As a consequence, the folding state and thus the knotted nature of the disulfides can easily be verified by detecting either formation of the respective fragments upon CID or *via* comparison of the ratios of the formation of loop 1 in the folded and unfolded state, which should be typically in the range of 10. Taking into consideration the reproducibility of this analysis, this methodology can be applied to the analysis of structure and disulfide bond connectivities of other miniproteins of the MCoTI family and may be useful for other peptides with related multidisulfide pattern and interesting pharmacological properties as e.g. cyclotides or conotoxines [17], [18]. The influence of tertiary structure on fragmentation patterns of MS/MS spectra may lead to information on the region of protein-protein interaction with ramifications for analysis of various biomolecular interactions as e.g. antibody-antigen binding or conformational changes of antigens upon antibody binding.

## Supporting Information

Figure S1 **Initial spectra of 1.** (**A**) MS^1^ of **1.** (**B**) Zoom-In on M+2H]^2+^. (**C**) Zoom-In on M+5H]^5+^.
(PNG)

Figure S2 **Initial spectra of unfolded precursor of 1.** (**A**) MS^1^ of unfolded precursor of **1.** (**B**) Zoom-In on M+2H]^2+^.
(PNG)

Figure S3 **Initial spectra of 2.** (**A**) MS^1^ of **2.** (**B**) Zoom-In on M+2H]^2+^. (**C**) Zoom-In on M+5H]^5+^.
(PNG)

Figure S4 **Initial spectra of unfolded precursor of 2.** (**A**) MS^1^ of unfolded precursor of **2.** (**B**) Zoom-In on M+2H]^2+^.
(PNG)

Figure S5 **Initial spectra of 3.** (**A**) MS^1^ of **3.** (**B**) Zoom-In on M+2H]^2+^.
(PNG)

Figure S6 **Initial spectra of unfolded precursor of 3.** (**A**) MS^1^ of unfolded precursor of **3.** (**B**) Zoom-In on M+2H]^2+^.
(PNG)

Figure S7 **MS^2^ of MCoTI peptide 2.** (**A**) CID of the triply charged ion of reduced precursor of peptide **2.** (**B**) CID of the triply charged ion of folded miniprotein **2.** (**C**) CID of the fivefold charged ion of folded miniprotein **2.**
(PNG)

Figure S8 **MS^2^ of MCoTI peptide 3.** (**A**) CID of the triply charged ion of reduced precursor of peptide **3.** (**B**) CID of the triply charged ion of folded miniprotein **3.**
(PNG)

Figure S9 **MS^3^ of major fragments of MCoTI peptide 2.** (**A**) MS^3^ of c_10_ ion. (**B**) MS^3^ of b_16_z_20_ ion. (**C**) MS^3^ of y_14_ ion. Arrows above the spectra indicate intensity amplifications.
(PNG)

Figure S10 **MS^3^ of major fragments of MCoTI peptide 3.** (**A**) MS^3^ of c_10_ ion. (**B**) MS^3^ of y_14_ ion. Arrows above the spectra indicate intensity amplifications.
(PNG)

Figure S11 **MS^3^ of y_14_-32 (A) and y_14_+32 (B) for the combinatorial interpretation of 2.** In red are inexistent peaks to provide evidence on the respective Ptc or Dha cleavage. Arrows above the spectra indicate intensity amplifications.
(PNG)

Figure S12 **MS^3^ of y_14_-32 (A) and y_14_+32 (B) for the combinatorial interpretation of 3.** In red are inexistent peaks to provide evidence on the respective Ptc or Dha cleavage. Asterisk indicates fragment from different parent ion with identical mass. Arrows above the spectra indicate intensity amplifications.
(PNG)

Figure S13 **MS^3^ of b_14_z_20_-32 fragment of MCoTI peptide 2.**
(PNG)

Table S1 **Applied source and gas parameters.**
(DOCX)

Method S1 **Experimental procedure for mass spectrometric measurements.**
(DOC)
