# Supplementary figures and images for: Fragmentation Follows Structure: Top-Down Mass Spectrometry Elucidates the Topology of Engineered Cystine-Knot Miniproteins
Source: PLoS One. 2014 Oct 10;9(10):e108626. doi: 10.1371/journal.pone.0108626 (PMC4193770; doi:10.1371/journal.pone.0108626)

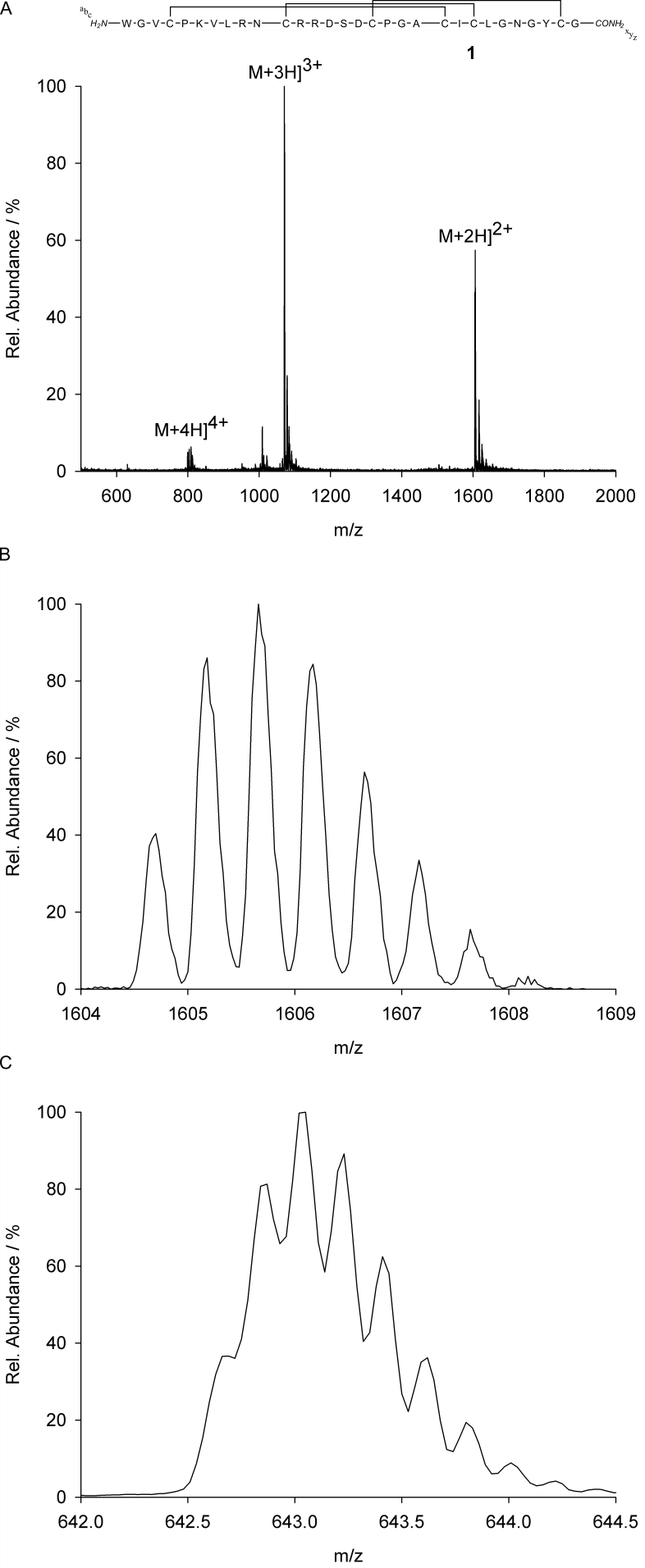

Supplement: Figure S1 — Initial spectra of 1. (A) MS1 of 1. (B) Zoom-In on M+2H]2+. (C) Zoom-In on M+5H]5+. (PNG) [file pone.0108626.s001.png]

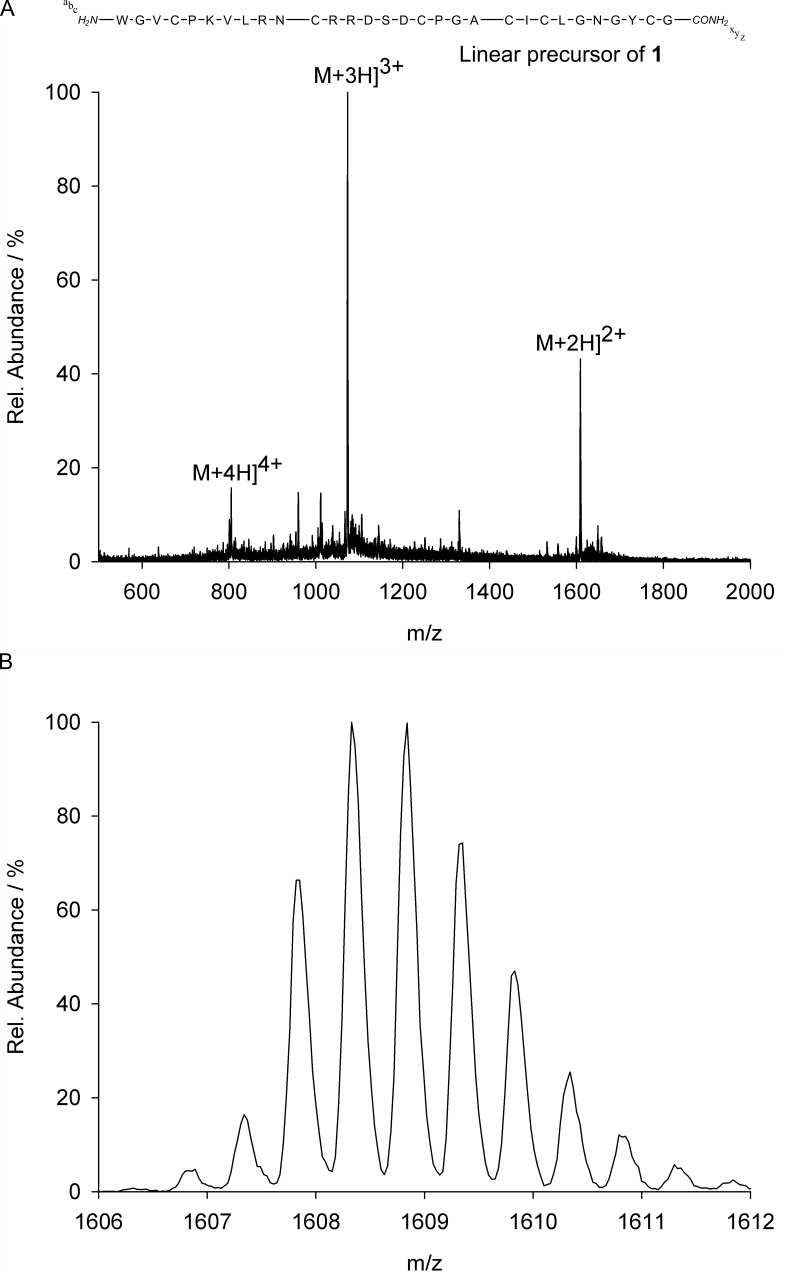

Supplement: Figure S2 — Initial spectra of unfolded precursor of 1. (A) MS1 of unfolded precursor of 1. (B) Zoom-In on M+2H]2+. (PNG) [file pone.0108626.s002.png]

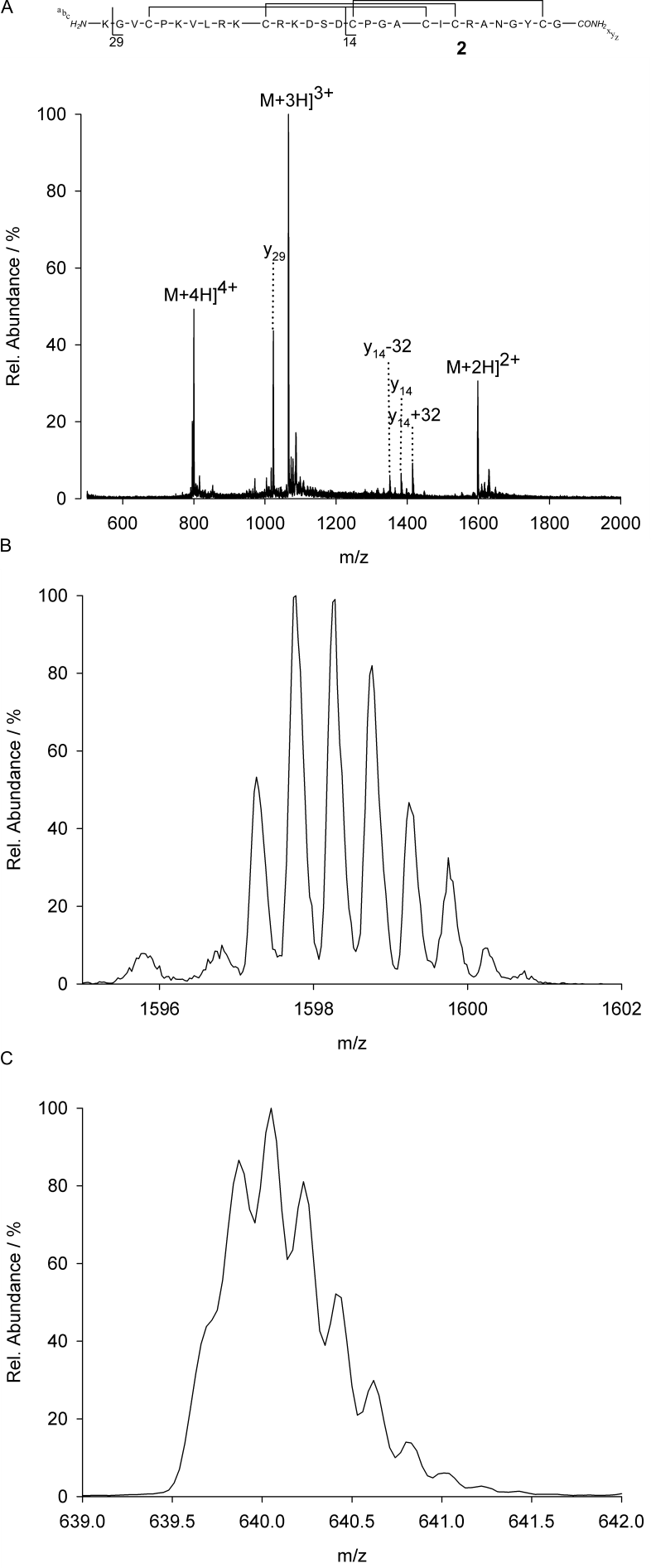

Supplement: Figure S3 — Initial spectra of 2. (A) MS1 of 2. (B) Zoom-In on M+2H]2+. (C) Zoom-In on M+5H]5+. (PNG) [file pone.0108626.s003.png]

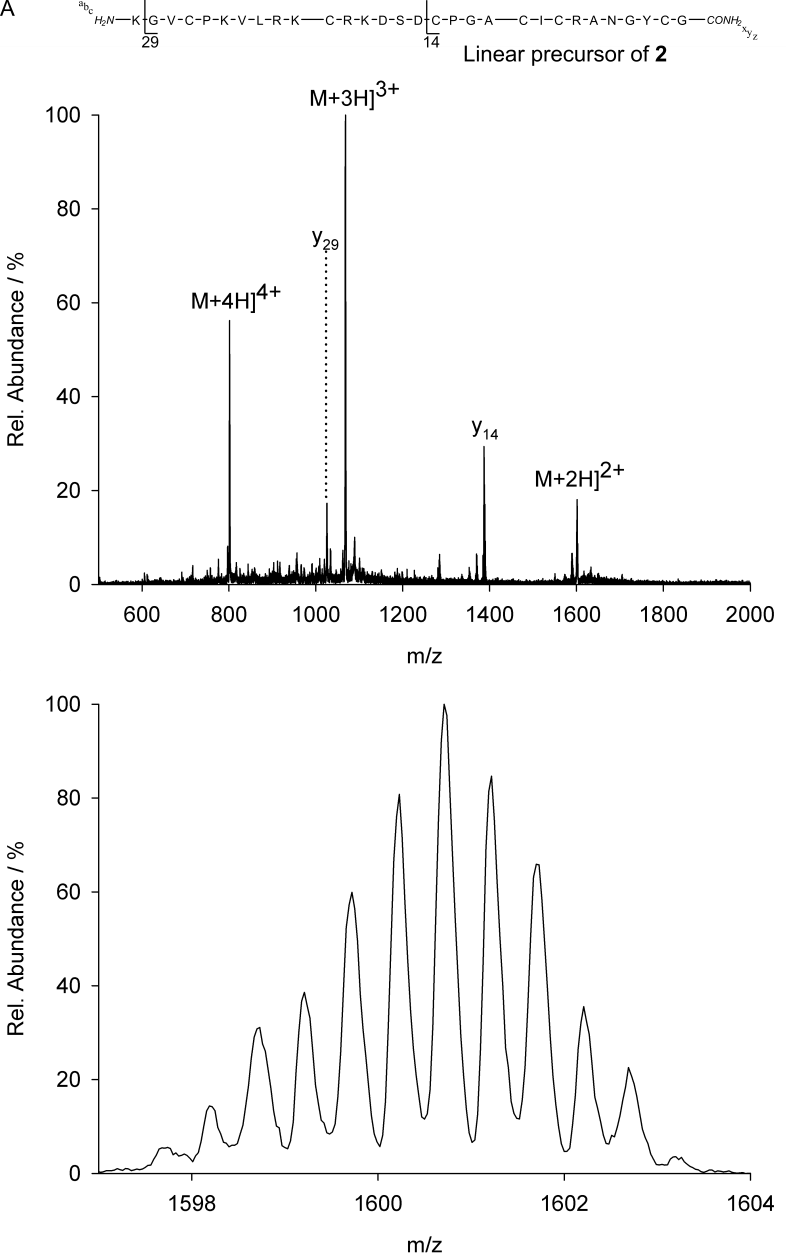

Supplement: Figure S4 — Initial spectra of unfolded precursor of 2. (A) MS1 of unfolded precursor of 2. (B) Zoom-In on M+2H]2+. (PNG) [file pone.0108626.s004.png]

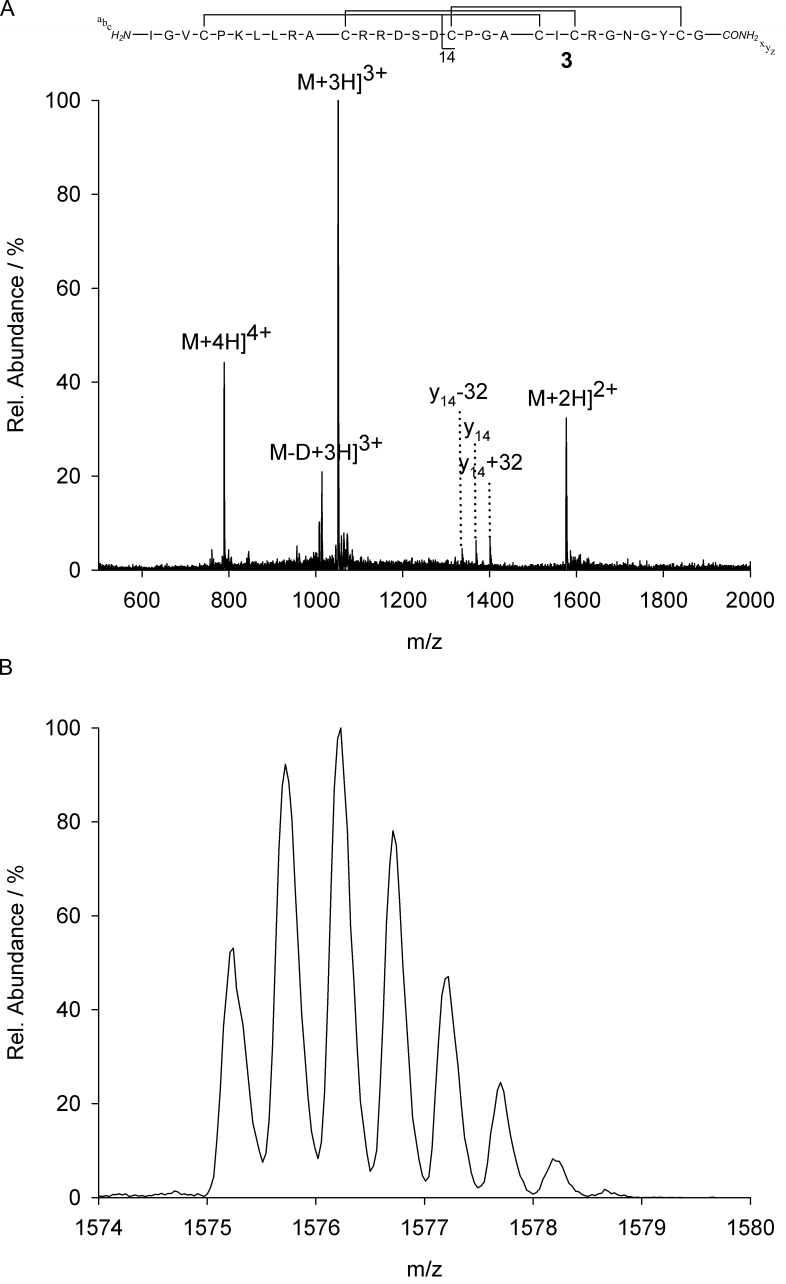

Supplement: Figure S5 — Initial spectra of 3. (A) MS1 of 3. (B) Zoom-In on M+2H]2+. (PNG) [file pone.0108626.s005.png]

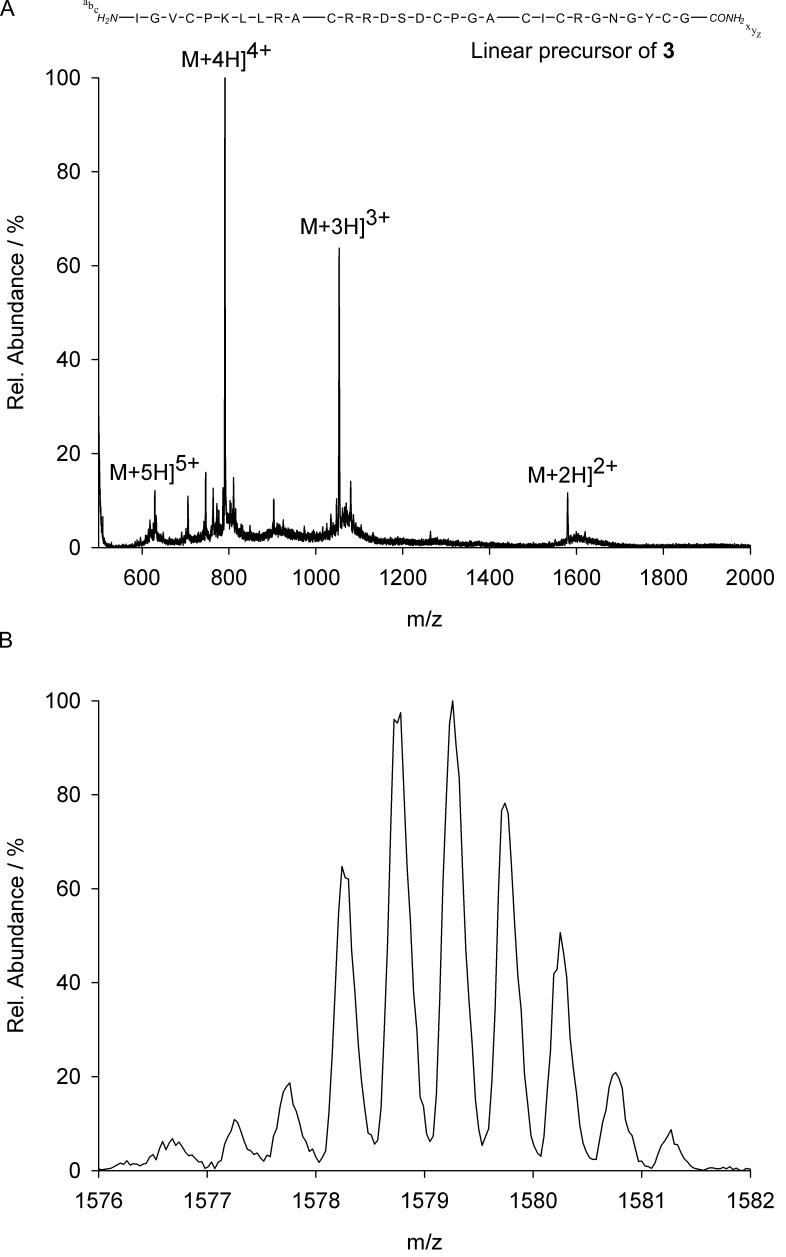

Supplement: Figure S6 — Initial spectra of unfolded precursor of 3. (A) MS1 of unfolded precursor of 3. (B) Zoom-In on M+2H]2+. (PNG) [file pone.0108626.s006.png]

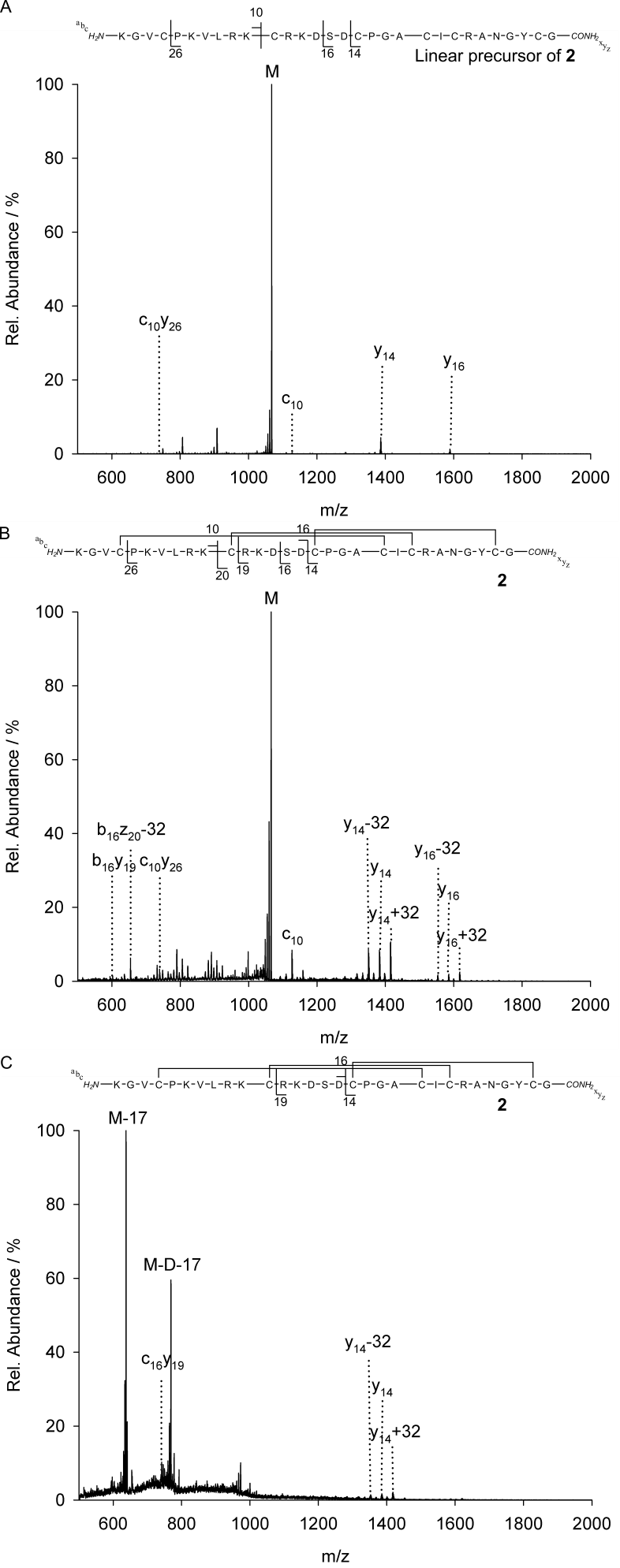

Supplement: Figure S7 — MS2 of MCoTI peptide 2. (A) CID of the triply charged ion of reduced precursor of peptide 2. (B) CID of the triply charged ion of folded miniprotein 2. (C) CID of the fivefold charged ion of folded miniprotein 2. (PNG) [file pone.0108626.s007.png]

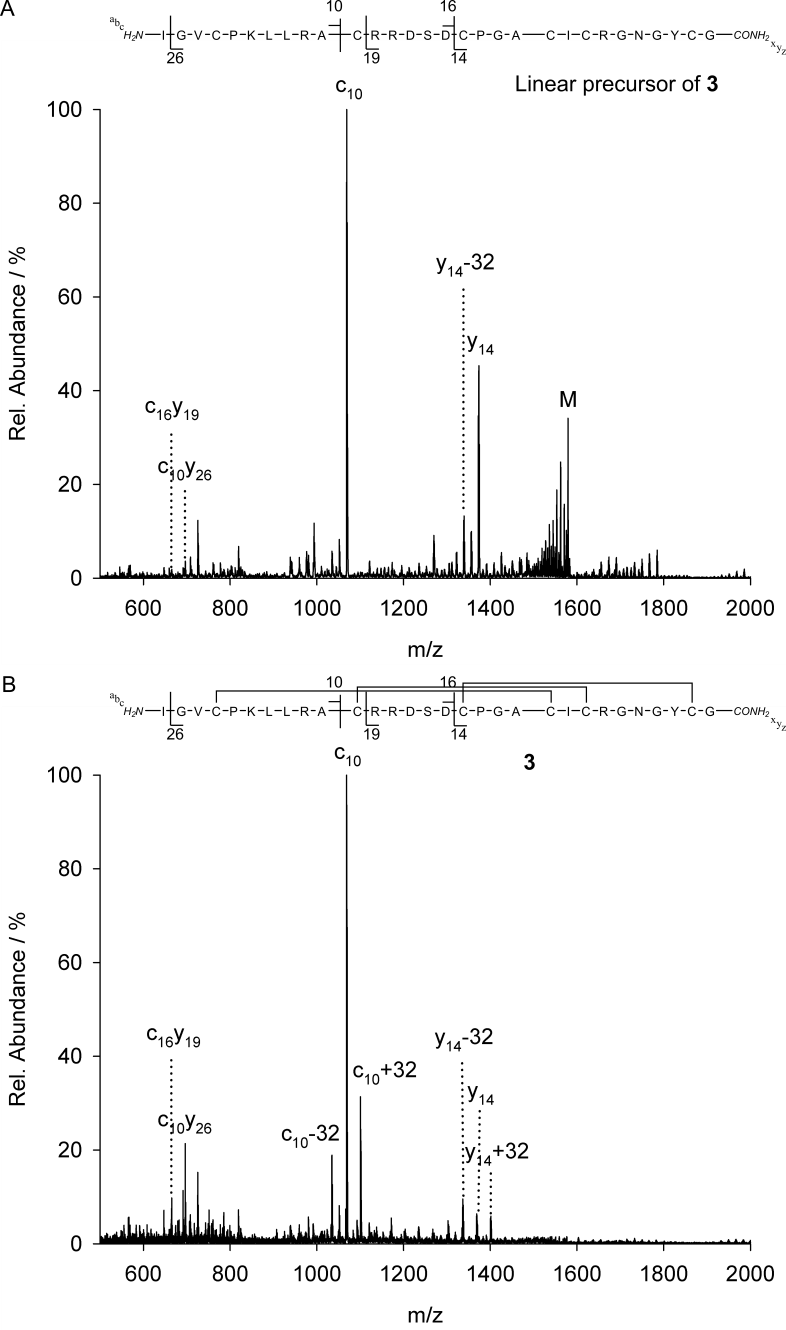

Supplement: Figure S8 — MS2 of MCoTI peptide 3. (A) CID of the triply charged ion of reduced precursor of peptide 3. (B) CID of the triply charged ion of folded miniprotein 3. (PNG) [file pone.0108626.s008.png]

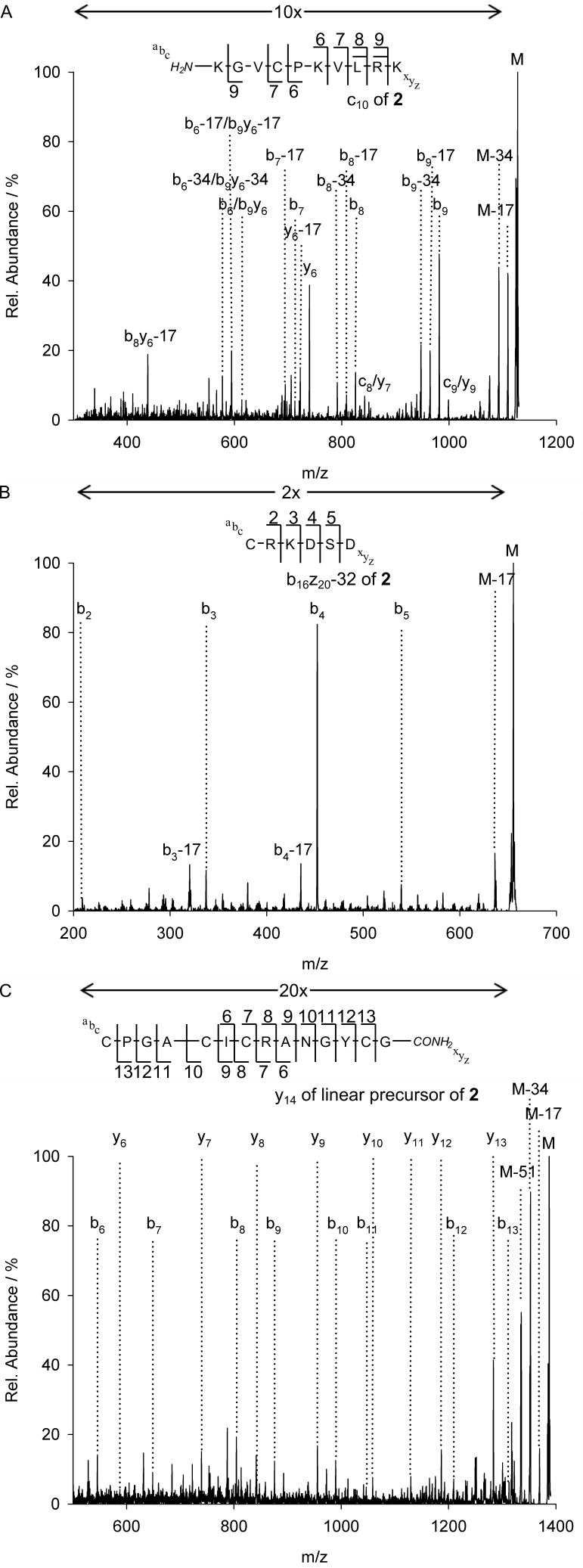

Supplement: Figure S9 — MS3 of major fragments of MCoTI peptide 2. (A) MS3 of c10 ion. (B) MS3 of b16z20 ion. (C) MS3 of y14 ion. Arrows above the spectra indicate intensity amplifications. (PNG) [file pone.0108626.s009.png]

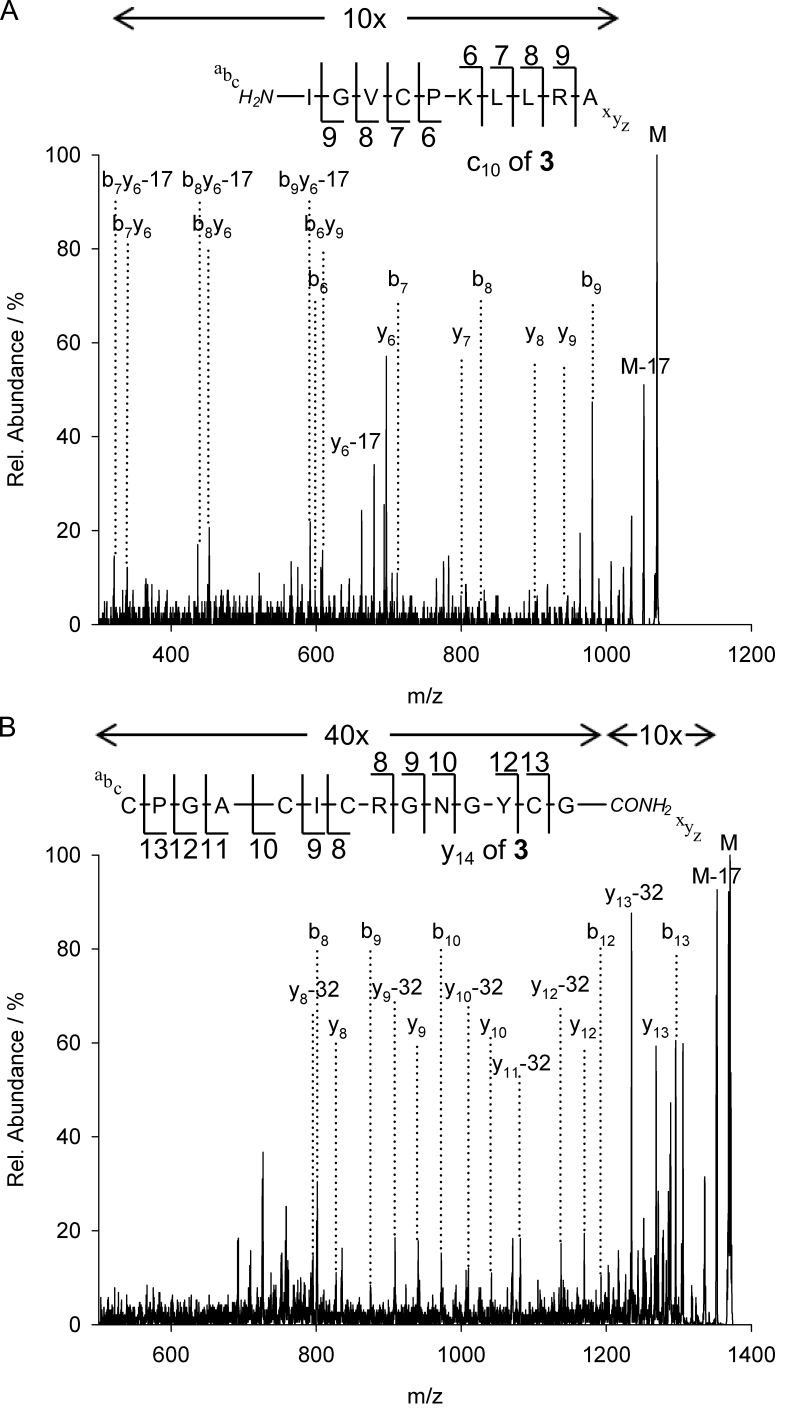

Supplement: Figure S10 — MS3 of major fragments of MCoTI peptide 3. (A) MS3 of c10 ion. (B) MS3 of y14 ion. Arrows above the spectra indicate intensity amplifications. (PNG) [file pone.0108626.s010.png]

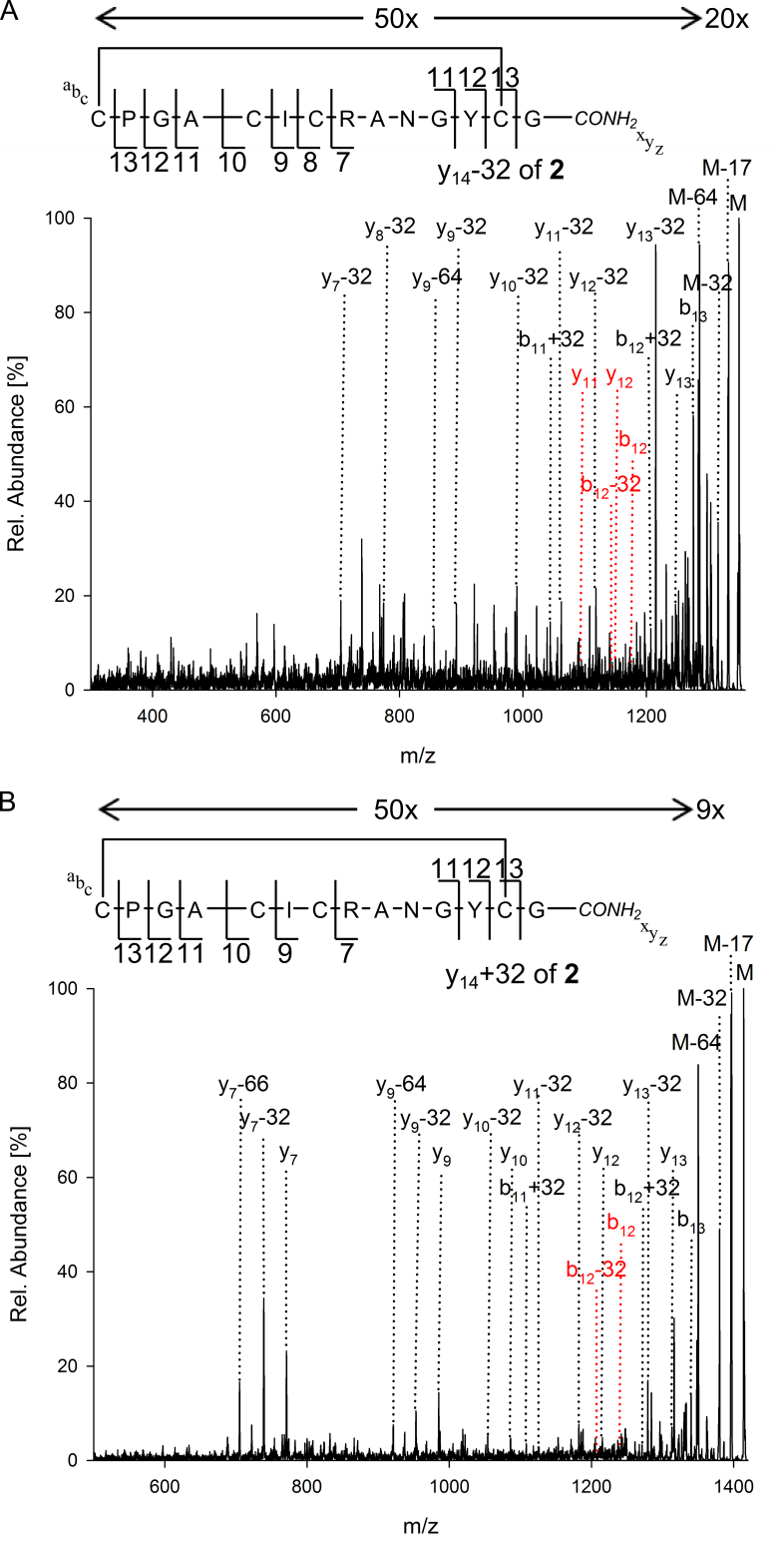

Supplement: Figure S11 — MS3 of y14-32 (A) and y14+32 (B) for the combinatorial interpretation of 2. In red are inexistent peaks to provide evidence on the respective Ptc or Dha cleavage. Arrows above the spectra indicate intensity amplifications. (PNG) [file pone.0108626.s011.png]

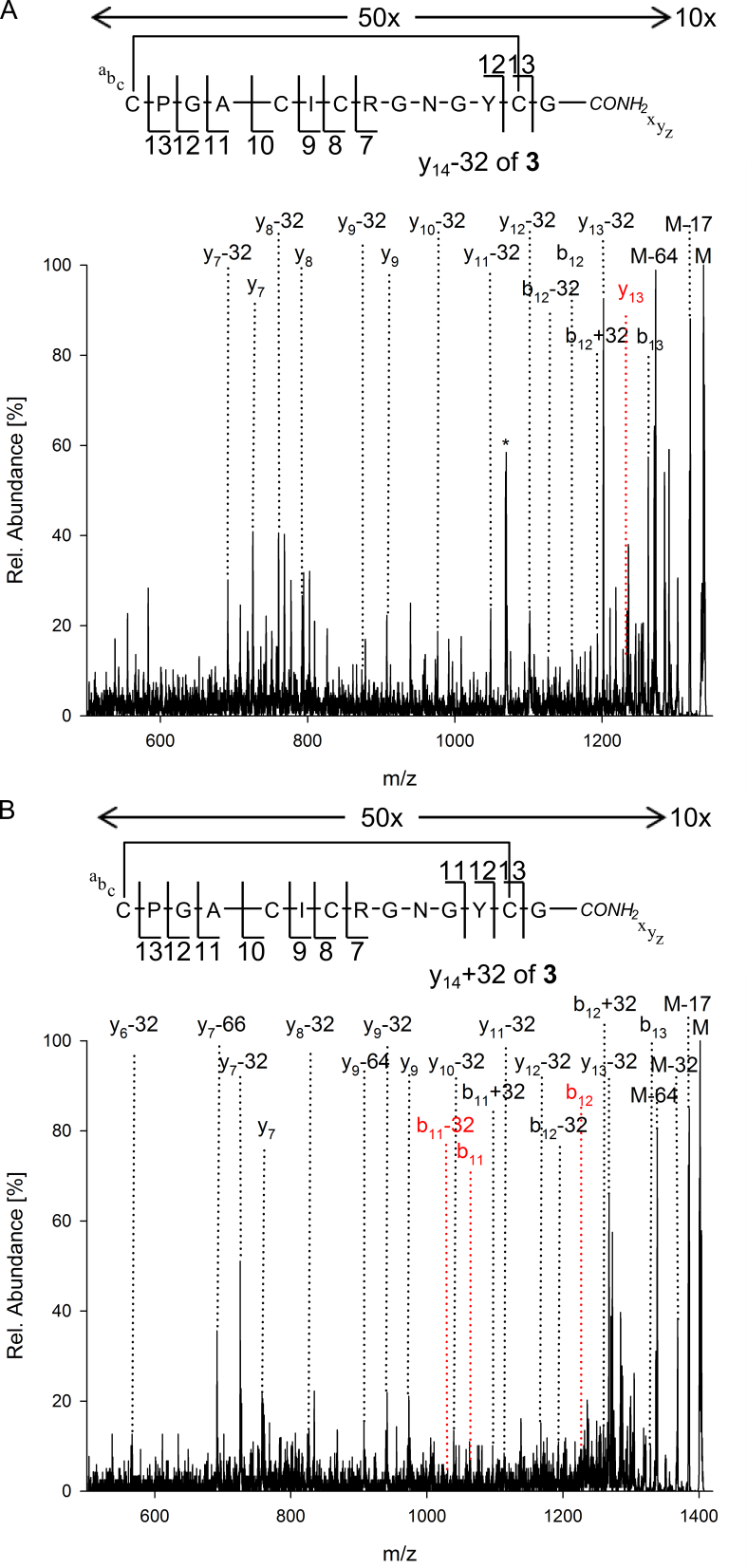

Supplement: Figure S12 — MS3 of y14-32 (A) and y14+32 (B) for the combinatorial interpretation of 3. In red are inexistent peaks to provide evidence on the respective Ptc or Dha cleavage. Asterisk indicates fragment from different parent ion with identical mass. Arrows above the spectra indicate intensity amplifications. (PNG) [file pone.0108626.s012.png]

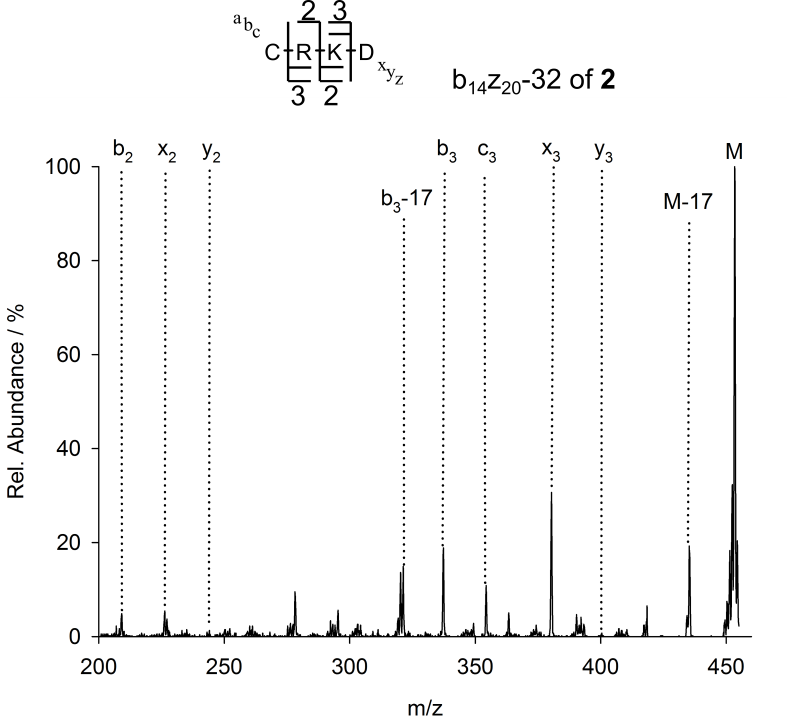

Supplement: Figure S13 — MS3 of b14z20-32 fragment of MCoTI peptide 2. (PNG) [file pone.0108626.s013.png]
