# Supplementary material for: Fragmentation Follows Structure: Top-Down Mass Spectrometry Elucidates the Topology of Engineered Cystine-Knot Miniproteins
Source: PLoS One. 2014 Oct 10;9(10):e108626. doi: 10.1371/journal.pone.0108626 (PMC4193770; doi:10.1371/journal.pone.0108626)
Supplement: Table S1 — Applied source and gas parameters. (DOCX) [file pone.0108626.s014.docx]

| **Table S1**: Applied source and gas parameters. | | |
| --- | --- | --- |
|  |  |  |
|  | **4000 QTRAP®** | **6500 QTRAP®** |
| Controll Software | Analyst 1.5.2 | Analyst 1.6.2 |
| Curtain gas (Cur) | 1.38 bar (20 psi) | 2.41 bar (35 psi) |
| Ionization Voltage (IS) | 5000 V | 5500 V |
| Temperature (TEM) | Room temp. | Room temp |
| Sprayer gas (GS1) | 1.03 bar (15 psi) | 0.89 bar (13 psi) |
| Heater gas (GS2) | 0 bar | 0 bar |
| Declustering  Potential (DP) | 80 V or ramped^*^ | 60 V or ramped^*^ |
| Entrance Potential (EP) | 10 V | 10 V |

^*^The declustering potential was semi-automatically ramped and the value resulting in a maximum overall intensity was chosen for the experiment
