# Supplementary material for: Fragmentation Follows Structure: Top-Down Mass Spectrometry Elucidates the Topology of Engineered Cystine-Knot Miniproteins
Source: PLoS One. 2014 Oct 10;9(10):e108626. doi: 10.1371/journal.pone.0108626 (PMC4193770; doi:10.1371/journal.pone.0108626)
Supplement: Method S1 — Experimental procedure for mass spectrometric measurements. (DOC) [file pone.0108626.s015.doc]

**Experimental procedure for mass spectrometric measurements**

**MS full scans experiments:** For the present study the 4000 QTRAP® LC/MS/MS and the 6500 QTRAP® LC/MS/MS systems (AB Sciex Germany GmbH) were used. Peptide stock solutions were prepared by dissolving 0.5 mg of the respective peptide in 1 mL of a mixture of 20 % methanol (Fluka Analytical, LC-MS CHROMASOLV) and 80 % water (Merck KGaA, LC-MS LiChrosolv) with 0.2 % formic acid (Fluka Analytical, analytic additive). The stock solutions were diluted to achieve 1e6 counts per second (cps) maximal signal intensity for full scan (m/z 500- 2000, 200 Da/s, unit resolution) experiments. The analyte solutions were infused (10 µL/min) into the masspectrometer using a syringe pump equipped with a 1 mL Hamilton syringe (internal diameter 4.61 mm). The peptide ions were generated using the positive mode electrospray ionization (ESI +). Source and gas parameters are provided in Table S-1.

**Enhanced resolution experiments:** Enhanced resolution experiments were performed to verify peptide charge states. The parameters resulting from the optimization of full scan experiments were used. However, the DP was semi-automatically re-optimized to yield a maximum intensity for the ion of interest. The first quadruple mass filter enabled ions ±15 Da of the target mass to pass into the linear ion trap. The optimal ion trap fill time was automatically determined (dynamic fill time, DFT) based on a total ion current target (TIC) of 0.5×1e7 cps (QT4000®) or 1×1e7 cps (QT6500®). To ensure an optimal resolution the ion trap scan was performed with a speed of 250 Da/s. A minimum of 100 scans were accumulated.

**MS/MS experiments:** As a starting point, the parameters resulting from the optimization of full scan experiments were used. However, the DP was semi-automatically optimized to achieve maximum intensity of the parent ion of interest. The collision energy was set by default to 50 V. If not appropriate the parameter was adjusted in 10 V steps or semi-automatically optimized *via* ramping. The optimal ion trap fill time for enhanced product ion scans was automatically determined (dynamic fill time, DFT) based on a total ion current (TIC) target of 2×1e7 cps (QT4000®) or 10×1e7 cps (QT6500®). Up to 500 scans were accumulated using maximal scans speeds of 4000 Da/s (QT4000®) or 10000 Da/s (QT6500®).

**MS^3^ Experiments:** For MS^3^ experiments the presetting from MS/MS measurements were used, while resonance excitation energy (AF2) was semi-automatically optimized (using 'ramping' functionality of Analyst software). For mapping of all MS^3^ fragments the smallest AF2 value allowing for full fragmentation of the parent fragment-ion was chosen. Regarding low-intensity fragments, AF2 energy was adjusted to achieve maximized intensities of those specific ions. Excitation times of 25 or 100 ms were used. The Q3 entry barrier was set to 8V.
